# Delay in Permanent Vascular Access Formation and Referral to a Nephrologist in Incident Hemodialysis Patients: A Single Center Experience

**DOI:** 10.7759/cureus.20728

**Published:** 2021-12-27

**Authors:** Natasha Khatri, Kiran Nasir, Murtaza Dhrolia, Ruqaya Qureshi, Aasim Ahmad

**Affiliations:** 1 Nephrology, The Kidney Centre Post Graduate Training Institute, Karachi, PAK; 2 Nephrology, The Kidney Center Post Graduate Training Institute, Karachi, PAK

**Keywords:** delayed refferal, maintenance hemodialysis, hemodialysis access, arteriovenous graft, arterio-venous fistula, central venous catheter (cvc)

## Abstract

Objective: This study assessed the factors associated with delayed referral to a nephrologist and delay in formation of a permanent vascular access in incident hemodialysis (HD) patients.

Methods: This prospective cross-sectional study was conducted from February 2021 to July 2021 on end stage renal disease (ESRD) patients receiving maintenance hemodialysis (MHD) at our center. Data were collected at the bedside during the HD session about a referral to a nephrologist, about when they were asked for permanent vascular access formation and the reason for the delay in its formation.

Results: Out of 296 patients recruited in our study, 168 (56.8%) were male and 128 (43.2) were female. The mean age was 53.5±15 years (minimum of 19 years and maximum of 90 years). The most common reason for refusal of making permanent vascular access [arterio-venous fistula (AVF) or arterio-venous graft (AVG)] was fear of pain in our patients 65 (43.3%) followed by the denial of the disease 32 (21.3%). Among the study subjects, 231 (78%) patients were referred to the nephrologist immediately or within one month of their diagnosis. Some 152 (51.4%) of the patients were not in favor of making AVF whereas 151 (51%) refused for starting HD, hence most of our patients 181 (61.1%) initiated HD in emergency by a central venous catheter (CVC).

Conclusion: Early referral should be done by primary care physicians (PCPs) for the timely management of CKD patients. As CKD is a progressive disease, it requires special attention by a nephrologist for adjustment of patient’s medications, timely follow-up, counseling, the early formation of AVF for HD, and planning for renal transplant. In our study, the majority of our patients initiated their HD via CVC because of the delayed visit to a nephrologist. Most patients were asked for AVF formation on the same day of presentation to our nephrology unit as they had advanced CKD (Stage 5) 134 (51.4%). Most patients in our study delayed AVF formation 152 (51.4%). With timely referral to a nephrologist, the nephrologist will be able to do better and repeated counseling about the disease, its progression, and the need for permanent vascular access for initiation of HD while patients and their families will get more time to make decisions.

## Introduction

Chronic kidney disease (CKD) is a leading cause of mortality and morbidity globally [1]. The burden of CKD is increasing in South East Asia and in Pakistan [2]. Awareness of CKD is limited, not only among the general population but referring physicians too, thereby increasing the need for hospitalization and mortality [3]. Around 25%-40% of CKD patients need renal replacement therapy (RRT) in the form of dialysis or renal transplant, soon after referral to a nephrologist, which leads to higher morbidity, mortality, hospital admissions, and worse long-term survival [4].

Vascular access is fundamental for carrying out hemodialysis (HD). There are three main types of vascular access: native arteriovenous fistula (AVF), arteriovenous graft (AVG), and central venous catheter (CVC), which includes tunneled CVC (tCVC) and non-tunneled CVC (ntCVC) [5]. The National Kidney Foundation-Kidney Disease Outcomes Quality Initiative (KDOQI) clinical practice guidelines for vascular access suggest that an AVF or an AVG is preferred to a CVC in most incident and prevalent HD patients due to the lower infection risk [6]. AVF is associated with lower mortality and morbidity [7-8].

The KDOQI also recommends an early formation of AVF when compared to AVG in incident HD patients to prevent vascular complications, like thrombosis, loss of primary patency, and interventions [6]. Incident HD patients with continuous use of CVC as their access experience reduced social life, poor sleep and energy, impact on daily routine, and reduced quality of life [9]. CKD patients showed more satisfaction with AVF as compared to other vascular access [10].

Most countries use CVC in more than 50% of the patients on initiating HD due to lack of patient’s knowledge of their disease, non-compliance to follow up, uncertain clinical presentation, and delayed referrals [11-12]. The incidence of CVC use is higher at the initiation of HD and the morbidity and mortality rate remains high in these patients [13]. Hence every effort should be made for the early formation of AVF. Primary care physicians (PCPs) refer CKD patients late to the nephrologist, which causes a delay in vascular access formation, initiation of HD with CVC, and delay in renal transplant [14-15].

This study was done to determine the frequency of CVC (ntCVC and tCVC) before permanent vascular access (AVG, AVF) is made, the time of referral to a nephrologist, and the reason behind the delay. This will help PCPs to understand that timely referral of CKD patients to a nephrologist is crucial for proper management, in choosing dialysis modality, counseling for permanent vascular access formation, and decision regarding renal transplant.

## Materials and methods

This prospective cross-sectional study was done at The Kidney Centre Postgraduate Training Institute, Karachi, Pakistan (TKC-PGTI) from February 2021 to July 2021 after approval from the hospital ethical review committee (TKC-ERC reference No. 113-NEPH-122020). Participants included in this study were adult end stage renal disease (ESRD) patients on maintenance hemodialysis (MHD) at our center, who willingly participated in the study by signing a written informed consent. Patients who received HD for acute kidney injury (AKI) and those with functioning renal transplant were excluded.

Data were collected on a pre-formed proforma, including age, gender, marital status, occupation, education, socioeconomic status, vascular access use at the time of initiation of HD, (tCVC, ntCVC, AVF, or AVG), and the reason behind the delay in getting permanent vascular access (AVF or AVG). The patients were asked about the time of diagnosis of CKD by PCP and when they were referred to a nephrologist after diagnosis.

Data were analyzed on IBM SPSS version 21 (SPSS Inc., Chicago, IL). Mean ± standard deviation was calculated for normally distributed continuous variables. Shapiro Wilk’s test checked the normality of data. Association of demographic and clinical variables with delay in the making of permanent access was established by chi-square test. The significant level was set as ≤ 0.05.

## Results

A total of 296 patients with a male to female ratio of 1.3:1 [males were 168 (56.8%) and females were 12 (43.2%)] participated in the study. The mean age of our study patients was 53.5±15 years (minimum of 19 years and maximum of 90 years). The majority of the study patients were married 260 (87.8%), while most patients 102(34.5%) were educated up to the primary level. Most of the male participants were unemployed 109(36.8%), while most of the female participants were stay-at-home mothers 109(36.8%). Most patients had a household income of PKR 50,000-100,000/month (300-600 USD) 107(36.1%). The most common comorbid in the study cohort was hypertension 284(95.9%) followed by diabetes mellitus 127(42.9%) (Table 1).

**Table 1:** Baseline characteristics of study patients (n= 296). ESRD, end-stage renal disease

Variables	n (%)	
Gender	Male	168(56.8)
	Female	128(43.2)
Marital status	Single	36(12.2)
	Married	260(87.8)
Education	Uneducated	67(22.6)
	Primary	102(34.5)
	Intermediate	29(9.8)
	Graduate	77(26)
	Post-graduate	21(7.1)
Employment status	Non-working	109(36.8)
	House wife	109(36.8)
	On job	46(15.5)
	Retired	32(10.8)
Monthly household income PKR (USD)	<25000 (<150)	35(11.8)
	25000-50000 (150-300)	132(44.6)
	51000-100,000 (300-600)	107(36.1)
	>100,000 (> 600)	22(7.4)
Comorbid conditions	Hypertension	284(95.9)
	Diabetes mellitus	127(42.9)
	Ischemic heart disease	45(15.2)
	Hepatitis	49(16.2)
	Hypothyroidism	18(6.1)
	Asthma	8(2.7)
	Cerebrovascular accident	5(1.7)
	Benign prostate hypertrophy	5(1.7)
Cause of ESRD	Unknown (CKDu)	128(42.3)
	Diabetic nephropathy	118(39.9)
	Glomerulonephritis	32(10.8)
	Renal stone disease	8(2.7)
	Adult polycystic kidney disease	8(2.7)
	Reflux nephropathy	1(0.3)
	Bilateral nephrectomy	1(0.3)

The most common reason for refusing permanent vascular access was fear of pain in our study participants 65(43.3%) followed by the denial of the disease 32(21.3%) (Table 2).

**Table 2:** Reasons of refusal of permanent access formation n (%).

Reasons of refusal	n (%)
Fear of pain	65(43.3)
Denial of disease	32(21.3)
Lifestyle disruption	16(10.7)
Bad experience of others	11(7.3)
Financial burden	10(6.7)
Lack of symptoms	5(2.3)
Family pressure	5(2.3)
Lack of vascular access knowledge	3(2)
Vascular tissue	2(1.3)
Dependency	1(0.7)

In the majority, the general physician made the diagnosis of ESRD 240 (81.1%). Most patients were referred to a nephrologist immediately or within one month of diagnosis 231(78%). The majority of the patients were asked for AVF formation on the same day of presentation to our nephrology unit as they have advanced CKD (Stage 5) 134(51.4%). Most of these patients were not in favor of making AVF or starting MHD 152(51.4%) and 151(51%) respectively, therefore, in the majority of patients, HD was initiated in the emergency department by the tCVC 181(61.1%) (Table 3).

**Table 3:** Parameters for initiation of HD n (%). ESRD, end-stage renal disease; HD, hemodialysis; AVF, arterio-venous fistula; CVC, central venous catheter

Variables		n (%)
Diagnosis of ESRD made by	General physician	240(81.1)
	Nephrologist	31(10.5)
	Other specialist	22(7.4)
	None	3(1)
Time of referral to nephrologist after diagnosis	Within 1 month	231(78)
	1 month-1 year	30(10.1)
	Self-referred	35(11.8)
When patient was asked to get permanent access	Same day	134(45.3)
	1 month-1 year	70(23.6)
	1-5 years	71(24)
	> 5 years	21(7.1)
Willingness for permanent access formation	No	152(51.4)
	Yes	144(48.6)
Willingness for initiation of HD	No	151(51)
	Yes	145(49)
Initiation of HD done in	Emergency	181(61.1)
	Elective	115(28.9)
Hemodialysis initiated by	AVF	115(38.4)
	CVC	181(61.1)
Time of delay in making permanent vascular access	No delay	122(41.2)
	1-3 months	78(26.4)
	4-6 months	56(18.9)
	7 months-1 year	25(8.4)
	> 1 year	15(5.1)

Age was significantly associated with delay in the formation of permanent access for MHD (p=0.018). Patients between ages 41 and 60 years did not delay AVF formation 72(59%) as compared to other age groups. Most unmarried patients got AVF earlier 26(72.7%) as compared to married patients, although the p-value was not significant (p= 0.08) (Table 4).

**Table 4:** Association of baseline factors of patients with delay in permanent access formation.

Factors of patients for delay in permanent access formation		Delay in permanent access formation		p value	
					No=122(41.2%)	Yes =174(58.8%)	
		Age of patients	≤ 40 years		18(27.7)	47(72.3)	0.018	
41 to 60 years	50(41)		72(59)				
> 60 years	54(49.5)		55(50.5)				
Gender	Male	72(42.9)	46(57.1)	0.511	
	Female	50(39.1)	78(60.9)		
Marital status	Single	10(27.8)	26(72.2)	0.08	
	Married	112(43.1)	148(56.9)		
Education	Uneducated	30(44.8)	37(55.2)	0.275	
	≤ Secondary	36(35.3)	66(64.7)		
	Intermediate	9(31)	20(69)		
	Graduation	36(46.8)	41(53.2)		
	Post-graduation	11(52.4)	10(47.6)		
Employment status	Non-working	41(37.6)	68(62.4)	0.46	
	Stay home mother	44(40.4)	65(59.6)		
	On job	20(43.5)	26(56.5)		
	Retired	17(53.1)	15(46.9)		
Monthly household income in PKR (USD)	< 25000 (<150)	14(40)	21(60)	0.31	
	25000-50000 (150-300)	55(41.7)	77(58.3)		
	51000-100,000 (300-600)	40(37.4)	67(62.6)		
	>100,000 (>600)	13(59.1)	9(40.9)		

## Discussion

As CKD patients have been increasing globally, the need for RRT is increasing. Timely referral to a nephrologist is essential for planning drug adjustment, lifestyle modifications, selection of modality of dialysis, vascular access formation for HD, initiation of dialysis, and decision about renal transplant. The KDIGO guidelines advise that the CKD patients should be referred to a nephrologist once the glomerular filtration rate (GFR) falls below 30 mL/min, but in many cases, the decision to initiate dialysis depends on patient’s symptoms like uremic encephalopathy, hyperkalemia, fluid overload, irrespective of the GFR [6,16]. 

Lack of symptoms, lack of awareness of the disease, fear of pain, denial of the disease, financial burden, and lifestyle disruption are the major patient-related factors [3], while lack of communication, poor working relationships, treating CKD patients on their own are physicians’ related factors in delayed referral to a nephrologist [17].

In our study, most patients were referred by general physicians 240 (81.1%). Among these patients, most visited a nephrologist first time when they already had CKD Stage 5. The majority were asked for permanent vascular access formation on the day of their first visit to a nephrologist 134 (45.3%). This delayed referral to a nephrologist was observed in many studies globally. Recently Dharod et al. reviewed records of 133,913 patients on follow-up by 185 primary care practices (61 practices). Out of these, 54.6% were not referred to nephrology [18]. Alfarhan et al. found that the most important physician and hospital factors are insufficient conduction of pre-dialysis care and education (63.7%) and late referral to a nephrologist (56.6%) [19].

Most of our study patients did not favor vascular access formation or the initiation of HD when diagnosed with ERSD 152(51.4%). The most common factor for refusal of permanent access formation was fear of pain in our study 65(43.3%), while 32(21.3%) denied their disease. Our results are similar to the study done by Alfarhan et al. [19] which showed denial in 76.4% and fear 75.9% as major causes of delay in permanent vascular access formation. 

Most of our study participants started HD in emergency via ntCVC 181(61.1%). Most of them delayed permanent vascular access formation for one to three months 78(26.4%) despite repeated counseling, while 56(18.9%) delayed it for four to six months. Similar results were found in a study done in Pakistan; it showed that 80% required CVC as their first access for MHD (96/120 patients), out of which 74.2% were dialyzed through ntCVC and 5.8% through tCVC [20].

Kim et al. concluded that 73.3% of CKD patients started dialysis with CVC, whereas 21.5% with AVF and 5.2% with AVG. The patients who initiated HD with AVF had a better outcome, high quality of life, low incidence of depression, and reduced hospitalization rate [7]. Locham et al. found that after adjusting for potential confounders, compared to AVF, patients with AVG [HR(95% CI): 1.35(1.31-1.40)] and HD using catheters (HC) [HR(95% CI): 1.80(1.77-1.84)] were more likely to develop sepsis at three years (both P < 0.001) [21].

Among all variables, age and marital status showed statistical significance in our study. Younger and older patients refused AVF more in comparison to middle age. Kim et al. found increased five-year mortality in association with CVC in CKD patients aged 65-74 or ≥ 75 years, as compared to <65 years [7]. Most of our married patients delayed AVF formation 148(56.9%) (P = 0.08). This could be a bias as the number of married patients was high as compared to unmarried in our study. Delay in AVF among married patients may be associated with family pressure.

A high level of satisfaction has been associated with the use of permanent vascular access, mainly with AVF in HD patients, which improves the quality of life and reduces mortality. Wasse et al. evaluated access use satisfaction in HD patients; out of 77 patients, 62.3% used AVF and were the most satisfied as compared to patients with tCVC 23.4% and AVG 14.3% [22].

Our study has a few limitations. It is a single-center study of MHD patients. The majority of the study patients were referred by PCPs late and at the time of need of initiation of HD. For this reason, most patients were asked to get AVF on their first visit, a decision of which was difficult for patients and their families for various reasons, including fear of pain and denial of the disease, leading to further delay in AVF formation. We did not compare the study cohort with patients on regular follow-up with our nephrology department.

## Conclusions

As CKD is a chronic progressive disease with high and rising incidence worldwide, awareness of the disease process is very important for CKD patients and their families by repeated counseling. PCPs should be more aware of early CKD diagnosis and should refer the CKD patients to a nephrologist early for drug adjustment, counseling, early permanent vascular access formation, and early planning of renal transplant. Early referral also helps in reduced use of CVC and hospitalization secondary to CVC-related complications.
